# Understanding political perceptions of tobacco policies and stakeholders in France: A qualitative study with parliamentarians

**DOI:** 10.18332/tpc/211970

**Published:** 2025-12-20

**Authors:** François Topart, Ana Millot, Emmanuelle Béguinot, Janet Hoek, Karine Gallopel-Morvan

**Affiliations:** 1 EHESP School of Public Health, University of Rennes, Rennes, France; 2 French National Committee for Tobacco Control, Paris, France; 3 Department of Public Health, University of Otago, Wellington, New Zealand

**Keywords:** tobacco industry, taxation, policies, parliamentarians, multiple streams framework

## Abstract

**INTRODUCTION:**

Characterized by persistently high smoking prevalence, France nonetheless faces difficulties in implementing coherent and sustained tobacco control measures. To understand what sustains this situation, we explored how French parliamentarians perceive tobacco, tobacco control policies (with a focus on taxation), and the stakeholders they believe should inform policy decisions.

**METHODS:**

As all parliamentarians vote on tobacco control policies, between March and June 2022 we undertook 25 individual interviews with French senators and deputies representing diverse political affiliations and regions. Interviews were analyzed using reflexive thematic analysis, guided by Grounded Theory. The section on tax increases was analyzed deductively. We used NVivo 15 for data management.

**RESULTS:**

Among the 25 parliamentarians (17 senators, 8 deputies), they acknowledged the health and economic harms of tobacco, although some minimized its risks. They were more likely to consider media/educational campaigns and taxation as effective, and tobacco sales ban or plain packaging as not. Most accepted anti-tax arguments promoted by the tobacco industry (illicit trade, ineffectiveness, regressivity), and were more likely to engage with tobacconists than health stakeholders during consultations.

**CONCLUSIONS:**

In line with streams identified in the Kingdon’s Multiple Streams Framework (problem, policy, political), this study suggests that competing framings of tobacco, weak support for evidence-based measures and a political environment favorable to the tobacco industry, weakened prospects for sustained tobacco control. Our findings highlight the need for further research exploring narratives likely to influence policymakers’ support for tobacco control measures.

## INTRODUCTION

Tobacco is a major preventable cause of premature mortality worldwide, responsible for the death of eight million people each year^1^. To counter this epidemic, the WHO Framework Convention on Tobacco Control (FCTC) outlines comprehensive evidence-based tobacco control measures^2^. Among these, the FCTC recommends raising taxes on tobacco products (Article 6), a policy known to reduce tobacco consumption^3^, and restricting tobacco industry lobbying (Article 5.3), which remains a major obstacle to tobacco control progress^4^.

Despite ratifying the FCTC in 2004, France has one of the highest smoking rates in Europe: in 2023, nearly a quarter of people aged 18–75 years smoked daily^5^. Smoking causes the premature death of 75000 people each year^6^, and imposes an estimated social cost of €156 billion^7^. To decrease prevalence, measures adopted in recent decades include excise tax increases, disallowing sales to minors, introducing an advertising ban (the Evin law, 1991), ending smoking in bars and restaurants (2006), requiring warnings (2010) and plain packaging (2016), developing regular campaigns (e.g. ‘Mois sans tabac’ held every November since 2016), and reimbursing nicotine replacement therapies (2019)^8^.These measures have decreased tobacco prevalence, but some policies lack enforcement (e.g. sales ban to minors, advertising ban), suffer from inconsistent approaches and poor coordination (e.g. media campaigns are launched without associated tax increases)^9^. As a result, prevalence is still high in France relative to the UK, Canada, and New Zealand, among other countries^10^.

Yet impediments undermining adoption of additional measures raise important questions about influences on parliamentarians’ support for effective tobacco control measures, particularly links with tobacco actors that oppose these measures^9^. Previous research found lobbying by tobacconists and tobacco industry (TI) may explain the inconsistent approach taken to excise tax increases in France^11-13^, which have alternated between periods of high tax increases (leading to decreased prevalence) and smaller increases or tax freezes (with correspondingly little or no effect on consumption)^14^.

To better understand French tobacco control barriers, we interviewed 25 Deputies and Senators, who play an important role in drafting and voting on policies, to probe their perceptions of tobacco in general and its impact on individuals and French society. We also explored perceptions of tobacco control policies, particularly excise tax increases, identified as an effective measure strongly opposed by the TI in France^11-13^. Finally, we probed the stakeholders they engage with when making decisions on tobacco control measures (e.g. manufacturers, tobacconists, public health actors).

Kingdon’s Multiple Streams Framework (MSF) provides a lens for understanding the insights gained from the interviews^15^. It examines the policy-making process, particularly why some policies proceed and are passed into law while others do not. Specifically, the MSF suggests political decision-making results from three streams. The ‘problem stream’ first considers how situations come to policymakers’ attention and then move up the hierarchy of problems requiring political action. Second, the ‘policy stream’ concerns solutions proposed by ‘experts’ (interest or advocacy groups) to address the identified problem. Finally, the ‘political stream’ encompasses the broader political context, ‘composed of such things as public mood, pressure group campaigns, election results, partisan or ideological distributions in Congress and changes of administration’^15^. The convergence of these three streams at critical time points creates a window of opportunity during which problems reach the political agenda and policy decisions may occur.

Our research aims to identify obstacles France faces in significantly reducing smoking prevalence, and thus why the window of opportunity does not seem to occur.

## METHODS

### Design

Qualitative research aims to provide in-depth insights into complex phenomena, and gathers participants experiences, perceptions, and behavior^16^. To explore decision making processes, we conducted 25 individual in-depth interviews with French parliamentarians, between March and June 2022; interviews were either conducted in Paris or online. Excluding the section devoted to arguments on tax increases, which is based on a deductive approach (Policy Dystopia Model)^17^, we used an inductive approach and interpreted the data using a reflexive thematic analysis^18^. Guided by Grounded Theory in the analysis^19^, we later interpreted the results of the 25 interviews through the lens of the MSF. Our study followed the qualitative research review guidelines (RATS).

### Sample

Participants were parliamentarians (17 Senators and 8 Deputies), whose role involves tobacco control policy making (see Supplementary file for details on French legislative process). Participants varied in their age, geographical base, and political affiliation. Table 1 summarizes respondents’ profiles (see Supplementary file for further details).

**Table 1:** Characteristics of French parliamentarians interviewed in a qualitative study, Paris, March–June 2022 (N=25)

*Characteristics*	*n*
**Gender**	
Male	14
Female	11
**Age** (year)	
35–45	2
45–55	5
55–65	11
>65	7
**Region** ^a^	
Border regions	6
Interior regions	19
**Political affiliation**	
Left	7
Centre	14
Right	4

a French border regions are more affected by tobacco cross-border trade^20^. A previous study indicates stronger parliamentary activity on tobacco taxation among parliamentarians from these regions^11^.

**Figure 1:**
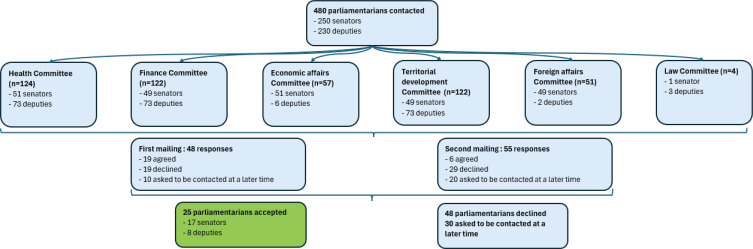
Flowchart of the recruitment process of French parliamentarians for qualitative interviews, Paris, March–June 2022

All current French deputies and senators were eligible to participate. Potential participants were contacted using their publicly available email addresses listed on the websites of the French National Assembly and Senate (https://www.assemblee-nationale.fr; https://www.senat.fr). We initially targeted members of the health and finance committees (146 deputies and 100 senators), believing they may be potentially more likely to agree to an interview, before expanding to other committees. In total, we contacted 230 deputies (out of 577) and 250 senators (out of 348) (Figure 1), representing 51.9% of French parliamentarians.

Our contact email explained that we were conducting academic research into decision-making regarding alcohol and tobacco policy (results on alcohol are presented elsewhere) and invited each recipient to participate.

We received 48 responses: 19 people agreed to participate, 19 declined, and 10 asked to be contacted later. Excluding those who already accepted or refused, we sent a follow-up email to 442 parliamentarians between 7 and 14 days after the initial contact. This second round yielded 55 responses: six agreed to participate, 29 declined and 20 asked to be contacted later. Recruitment ended once data saturation was achieved, after 25 interviews.

### Data collection

The interview guide explored participants’ views through open questions that probed:

1. How parliamentarians saw the risks and benefits of tobacco, both to individuals and society.
2. Which tobacco control measures they considered most effective, with a focus on tobacco taxation. After they gave their general opinion on taxation, we exposed parliamentarians to arguments highlighted in the Policy Dystopia Model (PDM) and three studies conducted in France^11–13,17^. These provided a taxonomy of political strategies that tobacco companies use to prevent tax increases (7 arguments), and also included arguments made by public health actors to promote tax increases (3 arguments) (Table 2).
3. How they perceived stakeholders involved in implementing tobacco control policies: the tobacco industry, tobacconists, health actors (public, NGOs). We also probed parliamentarians’ awareness of the WHO FCTC and Article 5.3, which obliges Parties to ‘*protect [its] policies from* … *interests of the tobacco industry*’^2^.

**Table 2:** Arguments in favor of or against high tax increases (based on the PDM model) presented to French parliamentarians interviewed, Paris, March–June 2022

*Domain*	*Argument*	*References*
**Tobacco economic actors’ arguments opposed to tobacco taxation**		
**Costs to economy and society**	Tax increases encourage parallel markets and/or illicit trade	11-13,17,23
	Tax increases penalize less well-off smokers	11-13,17,23
**Unintended benefits to undeserving groups**	Tax increases encourage insecurity and benefit parallel economy actors	11-13,17
	The aim of tax increases is to fill the state coffers	11-13,17
**Intended public health benefits will not be realized**	Without tax harmonization, tax increases are ineffective	11-13
	Tax increases must be limited (to 5% or 6%)	11-13
**Expected tobacco industry costs**	Tax increases have negative economic consequences for tobacconists	11-13,17,23
**Public health actors’ arguments in favor of tobacco taxation**		
**Benefits to economy and society**	Tobacco tax increases boost tax revenues	11-13,17,23
**Intended public health benefits**	Tax increases protect young people and the underprivileged	11-13
	Tax increases are an effective tool for reducing tobacco consumption	11-13

We conducted interviews via videoconference (18), telephone (6), and in-person (1), according to respondents’ preferences. Interviews lasted from 34 minutes to 1 hour and 56 minutes, with an average duration of approximately one hour.

Prior to each interview, all participants provided oral consent to participate and agreed that the interview could be recorded. Following each interview, participants signed a consent form allowing use and analysis of the data (in anonymous form). Data collection was conducted in compliance with point 35 of European General Data Protection Regulation ‘RGPD’, and with article 64 of French law on informatics and freedom^21^. The study protocol strictly followed the principles of the Declaration of Helsinki and those outlined by the French Data Protection Authority guidelines^22^.

### Data analysis

We transcribed and anonymized the audio recordings and then managed the data with NVivo 15 (Lumivero), using thematic content analysis.

## RESULTS

### Perceptions of tobacco’s risks and benefits

We began interviews by asking participants what the word ‘tobacco’ evoked for them. Each participant noted the harmfulness of smoking, and many referred to cancer and the risks of passive smoking. One-third illustrated these negative perceptions by citing a personal experience, often related to the illness or death of a relative:

*‘We all know people around us who had throat or lung cancer because they were smokers.’ (Senator, 55–65 years, male)*

Most also noted the addictiveness of tobacco products and the difficulty of quitting. Around half referred to costs smoking imposes on the health system and outlined inequities smoking creates, given its greater impact on people of lower socioeconomic status.

Yet, as well as recognizing these harms, almost a third minimized addictiveness and suggested that ‘low’ tobacco consumption (e.g. one cigarette a day) did not present serious health risks:

*‘The real problem isn’t someone who smokes a cigarette after dinner once a day.’ (Senator, 55–65 years, female)*

Furthermore, a quarter associated tobacco with pleasure, conviviality (e.g. cigars), and suggested tobacco was a component of French culture, or represented a human desire for vice or excess:

*‘I don’t agree with those who say that there is no pleasure in smoking. I find smoking pleasant.’*

(Senator, 55–65 years, male)

### Tobacco control measures which were considered most effective

We next asked parliamentarians which tobacco control measures they thought most and least effective. Participants regarded mass media and educational campaigns, and tobacco tax increases to be (or to have been) the most effective measures for reducing smoking, with around half citing these options (though a very small minority felt tax increases were the least effective measure):

*‘Prevention campaigns and increasing the price of tobacco … are the most effective.’* (Senator, 45–55 years, male)

A quarter suggested smoke-free places and smoking cessation assistance would reduce smoking prevalence, and a fifth considered that less harmful alternatives, especially electronic cigarettes, could help reduce smoking. Despite identifying policies that they thought could reduce smoking, parliamentarians also felt some existing measures had been ineffective. For example, almost a quarter thought graphic warnings and plain packaging, implemented in France in 2017, had had no effect on smoking behavior:

*‘The disgusting images … had absolutely no effect on consumers.’* (Senator, 55–65 years, female)

A third opposed what they saw as ‘prohibitive measures’ mostly referring to a tobacco sales ban:

*‘Prohibition, ... in my opinion, is the most disastrous option.’* (Senator, >65 years, male)

Second, we exposed participants to the seven anti-tax arguments identified in the PDM and presented in Table 2. All parliamentarians believed tobacco taxes would encourage illicit trade, and almost all believed cross-border purchases would increase:

*‘[Concerning] tobacco, we raised the pack price, … it doubled or tripled smuggling.’* (Deputy, 55–65 years, female)

This argument segued into three main concerns. First, almost all participants considered growth in parallel markets would penalize tobacconists; they anticipated challenges for tobacconists operating on or near borders, who they thought would face closure. They also anticipated the implications would go beyond retailers and suggested communities’ social life would be reduced:

*‘In rural areas, [tobacconists] contribute ... to social life and to life in general ... It’s a real concern for tobacconists.’* (Senator, >65 years, female)

Second, almost half believed that this increase in illicit trade would promote the parallel economy, including organized crime:

*‘Taxes are increasing, … consequently, this facilitates the mafia’s activities. If there is one economic sector that is thriving today, it is the organized crime.’* (Senator, >65 years, male)

Third, a third of participants associated growth in illicit trade with increased health risks, and viewed illicit tobacco as more harmful than legal tobacco:

*‘[Illicit cigarettes] are the most trafficked and … have almost nothing in common with regular cigarettes. It’s … very toxic, even more toxic than legal cigarettes.’* (Deputy, 45–55 years, female)

Despite these potential negative outcomes, a small minority suggested tobacconists could develop alternative revenue streams by diversifying their product range. Some also noted measures that could curb parallel markets:

*‘Either, on the one hand, further control the trunks of cars returning from these countries, further restrict the number of packages that can be purchased for personal consumption.’* (Deputy, >65 years, male)

Beyond concerns over parallel markets, most participants thought it was self-evident that an excise tax increase would see government revenue increase. Indeed, one third believed tax increases aimed to generate state income:

*‘Tobacco is a cash cow.’* (Senator, 55–65 years, female)

However, others viewed tax policies as primarily having health objectives.

Nonetheless, two thirds agreed that ‘tax increases penalize less well-off smokers’, which could lead people to forgo other purchases (food), or to turn to more harmful products (illicit tobacco, drugs). A small minority identified tobacco taxes as a ‘class policy’, likely to ‘prohibit’ less well-off people ‘from all pleasures’. However, others took a more utilitarian approach and argued that the outcome is worthwhile for people’s ‘own good’:

*‘The poorest also have the right to benefit from a public health policy.’* (Senator, >65 years, male)

Regarding the three pro-tax arguments presented in Table 2, participants had strikingly different views on the effectiveness of tax increases. Almost half saw this policy as efficient and called for regular increases:

*‘Once you decide to control tobacco through taxation, you have to make sure that the increases are applied regularly.’* (Senator, >65 years, female)

However, more than half thought taxes were ineffective or no longer effective, even among young people and less well-off people, due to the negative outcomes detailed above, or the low price-elasticity for addicts. They felt frustrated by persistently high smoking prevalence and wished to find other policies:

*‘I think we’ve reached the limit of the system.’* (Deputy, 55–65 years, male)

Participants held different views on effective taxation policy implementation. Half believed that only tax harmonization at the European level could reduce national consumption and some thought harmonization would reduce parallel markets in border areas. Participants’ views on the excise tax increases that should be introduced also varied. For example, a third favored limited increases to avoid unintended negative outcomes, though a similar proportion believed that only large tax increases would reduce smoking prevalence.

### Perceptions of tobacco stakeholders

We asked participants which stakeholders they felt should be consulted regarding tobacco control policy. Most considered that, as an elected representative, they had a responsibility to consult all stakeholders (health and economic), especially groups potentially affected by a policy. So long as stakeholders had an interest in a particular area, a small minority indicated they made ‘no distinction’ between them:

*‘I have never distinguished between the meetings I have held ... whether it was pro this or anti that.’* (Senator, >65 years, male)

A fifth believed that consulting with economic lobbyists or interest groups had no influence on their own decision-making and stressed they could differentiate between the general interests they had to represent and the particular interests these groups presented. Nonetheless, although almost half argued they adopted a ‘prudent’ attitude when speaking with lobbyists representing economic interests, they described these meetings as informative and helpful to their decision-making:

*‘There’s no shame in being around a table and everyone defending their position, present the issues, the difficulties and the improvements, and the workarounds.’* (Senator, 55–65 years, male)

Most participants thought it legitimate to meet with tobacco economic actors (manufacturers and tobacconists), which they described as ‘professional’ and ‘legal’ organizations. A quarter believed these discussions could help create health benefits, for example, by encouraging tobacconists to diversify their revenue streams, or supporting manufacturers to develop less harmful and addictive products:

*‘[Manufacturers] are there to make good quality products … So we need them to explain … how we can do better.’* (Deputy, 55–65 years, female)

However, despite those similarities, participants held differing views on economic actors. They thought it important to involve tobacconists in discussions, since these businesses would be affected by tobacco control policies. They also saw tobacconists as important to local communities, and suggested they created a network of local shops and public services that were essential to the economic and social life of the regions:

*‘Tobacconists, especially when … there is a lot of debate about the desertification of town centers, … must be a real actor in the consultation process.’* (Senator, 45–55 years, male)

However, almost half held negative opinions of tobacco manufacturers, and emphasized the need for vigilance in meetings, with some refusing to engage with them altogether. A third avoided meeting with tobacco company representatives for moral reasons, namely because they held them responsible for a deadly product and did not trust the information these companies provided:

*‘This tobacco industry is murderous, so in any case they have no legitimacy to intervene in the public policy decision-making process.’* (Deputy, 45–55 years, female)

Others noted more pragmatic reasons for not engaging with the TI, such as their limited importance in the domestic economy within their constituency.

Nearly three-quarters reported having met infrequently with tobacco economic actors (mostly with tobacconists, typically when tobacco taxation was on the parliamentary agenda). Initiated by members of the tobacco sector, these meetings focused on three general topics: tax increases, support for tobacconists, and parallel markets. To explore further the relationship between parliamentarians and tobacco economic actors, we asked participants about their knowledge of Article 5.3 (FCTC), which strictly limits engagement with tobacco companies during policy-making. None was aware of it.

One quarter of participants had met with public health representatives (tobacco control NGOs), considered them relevant information providers, and thought public health and tobacco control aims should guide their decisions:

*‘They need to be involved in virtually every stage of the process. Because they are there for the greater good of public health.’* (Senator, >65 years, male)

One-fifth considered that NGOs repeated well-known health arguments and ignored the economic and social consequences of their proposals, while policymakers had to consider these broader concerns:

*‘Their objective is not to know that ... rural areas are collapsing because … tobacconists have closed.’* (Deputy, 55–60 years, male)

A small minority stressed NGOs’ lack of democratic legitimacy and criticized their refusal to engage with the tobacco economic actors to develop a ‘collective’ decision.

## DISCUSSION

This research explores the impediments France faces in reducing smoking prevalence and identifies factors responsible for the country’s history of inconsistent tobacco control measures. To understand why the window of opportunity does not seem to occur in France, we use Kingdon’s MSF to discuss our results and analyze the weaknesses of each stream –problem, policy and political – that may hinder the emergence of policy opportunities.

Our findings suggest sustained ambiguities may undermine progressive policy making in France. Regarding the ‘problem stream’, parliamentarians saw smoking as harmful and addictive, yet also considered it pleasurable, convivial, and linked to individual freedom. These ambiguities became more complex when participants considered tobacco’s role, with some considering it essential to economic prosperity and social fabric (tobacconists). These framings are difficult to reconcile. As for the ‘policy stream’, participants questioned the effectiveness of evidence-based measures such as plain packaging or health warnings, privileging media and educational campaigns, despite the latter’s limited impact. While many acknowledged the potential impact of tax increases, they nonetheless supported tobacco industry’s arguments against taxation arguments identified in the Policy Dystopia Model, which describes how the industry frames taxes as penalizing a wide range of stakeholders and harming both the economy and society^17^. Concerning the ‘political stream’, participants reported twice as many contacts with tobacco sector representatives as with public health representatives and generally viewed tobacconists as a key economic and social actor to engage with in tobacco control. This stance may reflect their poor awareness of Article 5.3 of the Framework Convention on Tobacco Control (FCTC), which requires Parties to protect policies from TI interests.

Our results suggest that gaps within the problem, policy, and political streams hinder their convergence in France. The problem stream is weakened by the ambiguous perception of tobacco, which sustains framings promoted by tobacco economic actors that are detrimental to a public health paradigm. These competing framings prevent tobacco from being unanimously recognized as a ‘pressing problem’, thus limiting its prioritization on policymakers’ agenda^24^. The policy stream also shows fragility. Participants’ preference for limited evidence-based measures over stronger policies such as tax increases may reflect a greater salience of tobacco industry’s arguments over scientific evidence among policymakers^25^, undermining consensus on the viability and acceptability of data-driven measures required for their implementation. Notably, we identified significant barriers in the political stream. Combined with no awareness of Article 5.3, the institutional role of tobacconists in France (operating under the authority of the Ministry of Finance) and their privileged access to policymakers, entrenches the tobacco industry’s influence over policymakers through its close ties with tobacconists^26^. This power imbalance may affect the other streams by reinforcing industry-favored framings and shaping the policy options considered viable and politically acceptable. Together, these factors create a political environment favorable to the tobacco industry impeding the streams convergence.

Studies using the MSF to analyze tobacco control policy adoption offer insights into how strengthening the three streams could trigger political change that could be useful for public health actors in France. Regarding the problem stream, a Lebanese study highlighted that disseminating scientific evidence on the economic and social costs of tobacco to policymakers prevented minimization of tobacco as a problem^27^. Similarly, other studies using the MSF found that framing – whether in terms of health or budgetary issues – shaped policymakers’ adoption or rejection of a sugar-sweetened beverage tax in Colorado and Kansas^28^. These findings suggest that advocacy framings could be developed (e.g. outlining the tobacco financial, economic, and environmental impacts in France) to strengthen the problem stream. Within the policy stream, a study conducted in Mexico and Tennessee indicate that disseminating favorable public opinion surveys facilitated policymakers’ commitment to adopting smoke-free legislation^29^. In France, previous studies highlighted public support for tobacco tax increases. As public opinion is a significant force for policy change^30^, updated data might further strengthen this measure’s acceptability among policymakers. Concerning the political stream, a media campaign in Kentucky exposing the tobacco industry’s tactics reduced the impact of its arguments opposing smoke-free legislation^31^. Given the positive perceptions of tobacconists in France, highlighting their illegal practices (sales to minors) could affect their credibility among policymakers^32^.

Beyond this MSF literature that provides practical recommendations for public health actors, our research also offers theoretical contributions. Although the MSF has been used to examine tobacco control policy making in countries such as China, Mexico, Turkey and Iran^29,33-35^, it has not previously been used to for France or its specific attributes. Indeed, under the authority of the Ministry of Finance and maintaining close ties with the tobacco industry, tobacconists provide the tobacco sector with privileged access to public policymakers^12,36^. Because policymakers are rarely explored in research, our work offers insights into legislators’ perceptions, thus revealing obstacles that must be addressed to implement effective tobacco control measures.

### Limitations

Our study has limitations. Our sample was limited and did not include other influential actors such as ministers or senior civil servants. In addition, interviewer bias may have occurred, as both interviewers work in tobacco control. However, they conducted the interviews by adopting an open line of enquiry. Selection bias is also possible, since participants may have been more interested in public health than non-respondents. The diversity of viewpoints and the concerns voiced by some participants about the exclusion of the tobacco sector suggest that social desirability bias was limited, though it cannot be excluded. As a qualitative study, we did not aim for representativeness, though future research could attempt to quantify the beliefs we identified. Our study did not capture power dynamics between political actors. Future research could address this dimension using complementary methods such as focus groups or document analysis. Finally, interviews were initially analyzed by one researcher, but team discussions helped to test and refine views and ensured these were clearly linked to the data. Future work could build on our findings by undertaking experimental studies that compare policy framing and how these influence perceived acceptability among policymakers.

## CONCLUSIONS

France’s difficulties in implementing a set of coherent long-term tobacco control measures are reflected in a slow decline in smoking prevalence. The ambivalent views on tobacco held by parliamentarians, weak knowledge of the most effective and evidence-based tobacco control policies, and the positive image of tobacconists, may explain the non-convergence of streams required for political change. Further research on policymakers’ perceptions may help identify policy framings that could, in turn, inform an advocacy campaign and open a policy window that will enable adoption of progressive tobacco control measures.

## Supplementary Material



## Data Availability

The data supporting this research are available from the authors on reasonable request.
