# Supplementary material for: Understanding political perceptions of tobacco policies and stakeholders in France: A qualitative study with parliamentarians
Source: Tob Prev Cessat. 2025 Dec 20;11:10.18332/tpc/211970. doi: 10.18332/tpc/211970 (PMC12717828; doi:10.18332/tpc/211970)
Supplement: Supplementary file 1 [file TPC-11-60-s1.pdf]

The French Fifth Republic is a hybrid system, with characteristics of both a parliamentary and a presidential system. Executive power is held by the President of the Republic and the Prime Minister. The Prime Minister, appointed by the President of the Republic, is the head of government. The Prime Minister and the government are accountable to Parliament, which exercises legislative power in France. In France, Parliament is bicameral. It includes:

- The National Assembly, composed of 577 deputies elected for a five-year term by direct universal suffrage in a two-round, first-past-the-post system.
- The Senate, composed of 348 Senators elected for a six-year term by indirect universal suffrage, by a college of electors. Half the Senate's seats are renewed every three years.

Among the differences between these two chambers, two are particularly noteworthy. Firstly, in case of disagreement between the Assembly and the Senate on a text of law, the government can give the final word to the National Assembly. Secondly, unlike the National Assembly, the Senate cannot be dissolved by the government.

The National Assembly and the Senate each have eight permanent parliamentary committees, covering different areas of competence: economy, defense, health, finance, foreign affairs, etc. Deputies and Senators are equally divided between these eight permanent committees. One of the main functions of these committees is to prepare the legislative work in public session. With a few exceptions, bills and proposed legislation are debated in public session on the basis of the text adopted by the corresponding standing committee. However, while parliamentarians may be more involved in certain areas of expertise, depending on which parliamentarians' committee they belong to, laws are adopted in plenary session, i.e. voted on by all parliamentarians.

Each bill (submitted by the government) or proposed law (submitted by one or more parliamentarians) is voted on successively by the two assemblies. The legislative procedure is generally characterized by a back-and-forth movement of the text of the law between the two assemblies. Each of the two assemblies is then responsible for examining and, if necessary, modifying (amending) the text adopted by the other assembly. This passage from one assembly to another to reach a consensus is known as the "parliamentary shuttle". When a text is adopted in identical terms, it becomes law.

Tobacco taxation is discussed each year as part of the Social Security Financing Bill (PLFSS). As a bill, the PLFSS is submitted by the government to the National Assembly, and the text is then examined by the Senate. All parliamentarians are free to submit amendments on tobacco taxation. As with any other legislation, all parliamentarians are required to vote on the PLFSS, and therefore on tobacco taxation.

Code	Age	Gender	Region	Prof. sector	Position	Political commission	Political leaning	Int. modality
**1**	>65	M*	NB***	Medical sector	Deputy	Health	Centre	Videoconf.
**2**	55-65	F**	NB	Education	Deputy	Economy	Centre	Videoconf.
**3**	45-55	F	Borderer	Ministerial officer	Deputy	Finance	Left	Videoconf.
**4**	>65	M	NB	Engineer	Deputy	Finance	Centre	Phone
**5**	55-65	F	NB	Education	Deputy	Health	Centre	Videoconf.
**6**	55-65	M	NB	Politician	Senator	Finance	Centre	Videoconf.
**7**	55-65	M	NB	Education	Senator	Finance	Left	Videoconf.
**8**	45-55	F	NB	Entrepeneurship	Deputy	Finance	Centre	Videoconf.
**9**	>65	F	NB	Private sector	Senator	Health	Right	Videoconf.
**10**	45-55	M	NB	Private sector	Deputy	Sus. development	Centre	Phone
**11**	45-55	M	NB	Politician	Senator	Health	Right	Face-to-face
**12**	45-55	M	NB	Public sector	Senator	Finance	Left	Videoconf.
**13**	35-45	F	NB	Private sector	Deputy	Finance	Centre	Videoconf.
**14**	>65	F	NB	Agriculture	Senator	Health	Right	Videoconf.
**15**	55-65	M	NB	Agriculture	Senator	Economy	Left	Phone
**16**	55-65	M	Borderer	Agriculture	Senator	Economy	Left	Videoconf.
**17**	>65	F	Borderer	Medical sector	Senator	Health	Centre	Videoconf.
**18**	55-65	M	Borderer	Private sector	Senator	Health	Centre	Videoconf.
**19**	35-45	F	NB	Engineer	Senator	Finance	Right	Phone

**20**	55-65	F	NB	Civil servant	Senator	Health	Left	Videoconf.
**21**	55-65	M	Borderer	Politician	Senator	EU affairs	Centre	Videoconf.
**22**	>65	M	Borderer	Unknown	Senator	Finance	Centre	Phone
**23**	>65	M	NB	Engineer	Senator	Sus. development	Left	Videoconf.
**24**	55-65	F	NB	Law	Senator	Finance	Centre	Videoconf.
**25**	55-65	M	NB	Private sector	Senator	Finance	Centre	Phone

*Male
**Female
***Non borderer

© 2025 Topart F. et al.
